# Editorial: Microbial-driven carbon turnover from dry-wet cycling regions

**DOI:** 10.3389/fmicb.2024.1514941

**Published:** 2024-11-15

**Authors:** Peng Zhang, Min Luo, Chuancheng Fu, Leilei Xiao

**Affiliations:** ^1^Yunnan Provincial Key Laboratory of Soil Carbon Sequestration and Pollution Control, Faculty of Environmental Science and Engineering, Kunming University of Science and Technology, Kunming, China; ^2^College of Environment and Safety Engineering, Fuzhou University, Fuzhou, China; ^3^Marine Science Program, Biological and Environmental Science and Engineering Division (BESE), King Abdullah University of Science and Technology (KAUST), Thuwal, Saudi Arabia; ^4^CAS Key Laboratory of Coastal Environmental Processes and Ecological Remediation, Yantai Institute of Coastal Zone Research, Chinese Academy of Sciences, Yantai, China; ^5^The Yellow River Delta Ecological Research Station of Coastal Wetland, Chinese Academy of Sciences, Dongying, China

**Keywords:** aquatic ecosystems, greenhouse gases (GHGs), microbial metabolism, dry-wet cycling, carbon cycle

Aquatic ecosystems play a crucial role in the global carbon cycle, serving as both significant carbon sinks and as sources of greenhouse gases (GHGs). Dynamic redox fluctuations, driven by factors such as changes in water levels and periodic flooding, facilitate interactions among reduced substances, metal-bearing minerals, and oxygen (Nielsen et al., 2010). These interactions shape microbial communities and influence the dynamics of soil organic carbon (Lalonde et al., 2012; Xiao et al., 2023). While redox fluctuations impact carbon cycling processes driven by microorganisms, further research is needed to elucidate the specific mechanisms through which microbial species, metabolic pathways, and microbial residues affect soil carbon cycling (Hu et al., 2024; Luo et al., 2019). This Research Topic invites research on GHG emissions and carbon sequestration in areas characterized by dry-wet cycling, with a focus on microbial processes and their ecological impact on the carbon cycle. Topics of interest include innovative technologies for GHG mitigation and carbon storage, microbial adaptations to environmental changes affecting GHG emissions, mechanisms of microbial carbon fixation, and interactions between microbes, minerals, and carbon in aquatic ecosystems. The goal is to enhance our understanding of sustainable solutions for managing carbon dynamics in regions with moisture fluctuations.

In this Research Topic, the Research Topics encompass a variety of ecosystems, including wetlands, lakes, agricultural soils, estuarine intertidal zones, grasslands, and saltmarshes. The studies investigate the effects of plants, inorganic substances (e.g., inorganic nitrogen), drought, organic matter (e.g., phenolic compounds), iron-bearing minerals, precipitation, salinity and heavy metals on greenhouse gas (GHG) emissions, microbial metabolism, enzyme activities, and other relevant environmental factors.

Li T. et al. highlight the importance of soil enzymes in wetland carbon and nutrient cycling and their varied responses to drought. A meta-analysis of 55 studies shows that while most enzyme activities remain stable, phosphorus-related enzyme activity increases by 38% under drought conditions, possibly due to their greater resilience to phenolic biotoxicity. Long-term experiments confirm that drought-driven plant shifts lead to phenolic accumulation, limiting enzyme activity, with phosphorus-related enzymes being an exception. These findings highlight wetland ecosystems' adaptive responses to drought, offering insights for assessing drought impacts and guiding restoration efforts. Jiang et al. demonstrate that the invasive plant *Spartina alterniflora* significantly affects GHG production in tidal wetland sediments. The presence of this species increases the production rates of CH4 and CO_2_, as well as the levels of organic matter and the abundance of microbial genes in invaded areas. These effects vary with spatial and seasonal dynamics, showing higher GHG production in surface sediments (0–10 cm) during the summer months. This study underscores how the invasion of *S. alterniflora* modifies GHG dynamics in coastal wetlands, offering valuable insights into emissions and carbon storage potential. Similarly, Zhang et al. examine the impact of varying precipitation levels on soil CO_2_ emissions in a saltmarsh within the Yellow River Delta, China. Over a seven-year period, the results indicate that a 40% increase in precipitation enhances soil CO_2_ production potential by 66.2%, with the highest soil respiration rates occurring under these conditions. Additionally, the quantity and quality of soil organic carbon positively correlate with CO_2_ production, although microbial diversity remains unaffected by changes in precipitation. These findings suggest that moderate increases in precipitation may enhance CO_2_ emissions, thereby influencing the carbon sink function of these blue carbon ecosystems.

Li S. et al. discuss the effect of soil inorganic nitrogen (SIN) on the phylogenetic traits and ecological functions of bacterial communities in China's estuarine intertidal zones. The findings indicate that SIN significantly influences bacterial phylogenetic diversity and turnover, exhibiting spatial and seasonal variations. However, it shows only a weak direct relationship with nitrogen-cycling functional genes. Structural equation modeling reveals that SIN indirectly affects nitrogen metabolism potential by regulating bacterial phylogenetics, highlighting SIN's role in shaping microbial nitrogen cycling within estuarine wetlands. Ni et al. investigate the role of iron (Fe) oxides in the preservation and mineralization of organic carbon (OC) in wetland soils. Under anaerobic conditions, the addition of lignite (Lig) resulted in OC accumulation in both freshwater and saline-alkaline wetland soils, whereas the addition of hematite (Hem) led to significant OC loss, particularly in FW soils. Hematite inhibited microbial Fe(III) reduction, which enhanced OC mineralization by altering microbial community structures. In contrast, lignite promoted the formation of Fe-OC complexes, thereby supporting OC persistence. These findings suggest that crystalline Fe oxides primarily drive OC mineralization, while older, aromatic-rich carbon contributes to OC preservation, providing insights for wetland restoration and carbon sink recovery. Mazzoni et al. discuss microbialites in Bagno dell'Acqua Lake, which contain abundant calcium carbonate and silica. DNA sequencing of these microbialites reveals unique microbial communities that differ between emerged and submerged samples. Key phyla include *Proteobacteria, Actinobacteriota*, and *Bacteroidota*, with *Cyanobacteria* being most prevalent in the submerged layers. Microorganisms play a significant role in carbonate formation, with some capable of precipitating calcium carbonate and other minerals, suggesting their potential for phosphate recovery in low-phosphate environments. Wang et al. examine how copper (Cu) pollution in Panax notoginseng soil affects microbial metabolism and nutrient cycling. At low Cu levels (Cu100), soil organic carbon, total phosphorus, and microbial biomass increased, along with enhanced carbon source utilization. However, higher Cu concentrations reduced key enzyme activities, indicating nitrogen limitation and variable carbon use efficiency (CUE). Low Cu concentrations appear to enhance microbial carbon and nitrogen limitations, providing insights for managing metal-related environmental risks in China by 2035.

Looking ahead, the importance of aquatic ecosystems in the global carbon cycle is becoming increasingly prominent. As climate change and human activities intensify their impact on the environment, in-depth studies of the dynamics of microbial processes in ecosystems such as wetlands, lakes, and paddy fields will become crucial. Future research should focus on the effects of dynamic redox fluctuations on microbial communities and soil organic carbon, exploring the specific mechanisms through which different microbial species, metabolic pathways, and microbial residues influence soil carbon cycling. Additionally, developing innovative technologies to reduce GHG emissions and promote carbon storage will be key to achieving sustainable development. Research should also examine biological adaptations to environmental changes, particularly in regions characterized by dry-wet cycles, to enhance the functionality of carbon sinks. As our understanding of heavy metals in soil environments and their effects on microbial metabolism deepens, future management strategies will become more targeted to address the challenges posed by metal pollution. In summary, future research will contribute valuable scientific knowledge for combating climate change and provide feasible solutions for the conservation and restoration of aquatic ecosystems.
